# *catena*-Poly[[diaqua­copper(II)]-μ-7-oxa­bicyclo­[2.2.1]heptane-2,3-dicarboxyl­ato]

**DOI:** 10.1107/S1600536809000270

**Published:** 2009-01-10

**Authors:** Yun-Yun Wang, Rui-Ding Hu, Yan-Jun Wang

**Affiliations:** a Zhejiang Key Laboratory for Reactive Chemistry on Solid Surfaces, Institute of Physical Chemistry, Zhejiang Normal University, Jinhua, Zhejiang 321004, People’s Republic of China, and, College of Chemistry and Life Science, Zhejiang Normal University, Jinhua 321004, Zhejiang, People’s Republic of China

## Abstract

In the crystal structure of the title compound, [Cu(C_8_H_8_O_5_)(H_2_O)_2_]_*n*_, the Cu(II) cation is in a Jahn–Teller distorted six-coordination by two O atoms from water molecules, by the bridging O atom from the bicyclo moiety, by two carboxylate O atoms from two different carboxylate groups and by one carboxylate O atom from a symmetry-related bridging ligand.The polymeric structure is made up from double-strands propagating parallel to the *c* axis that are held together *via* inter­molecular O—H⋯O hydrogen bonds.

## Related literature

For related literature, see: Yin *et al.* (2003 ▶). 
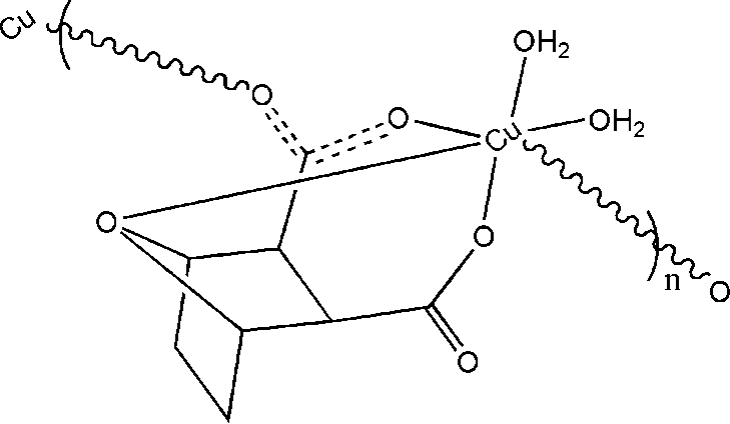


## Experimental

### 

#### Crystal data

[Cu(C_8_H_8_O_5_)(H_2_O)_2_]
*M* *_r_* = 283.72
Orthorhombic, 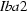

*a* = 10.5512 (4) Å
*b* = 19.3389 (9) Å
*c* = 9.7435 (4) Å
*V* = 1988.15 (14) Å^3^
*Z* = 8
Mo *K*α radiation
μ = 2.22 mm^−1^
*T* = 296 (2) K
0.29 × 0.20 × 0.12 mm

#### Data collection

Bruker APEXII area-detector diffractometer
Absorption correction: multi-scan (*SADABS*; Sheldrick, 1996 ▶) *T*_min_ = 0.60, *T*_max_ = 0.78
7372 measured reflections
2078 independent reflections
1897 reflections with *I* > 2σ(*I*)
*R*_int_ = 0.021

#### Refinement

*R*[*F* ^2^ > 2σ(*F* ^2^)] = 0.022
*wR*(*F* ^2^) = 0.059
*S* = 1.02
2078 reflections
157 parameters
9 restraints
H atoms treated by a mixture of independent and constrained refinement
Δρ_max_ = 0.32 e Å^−3^
Δρ_min_ = −0.38 e Å^−3^
Absolute structure: Flack (1983 ▶), 857 Friedel pairs
Flack parameter: 0.001 (16)

### 

Data collection: *APEX2* (Bruker, 2004 ▶); cell refinement: *SAINT* (Bruker, 2004 ▶); data reduction: *SAINT*; program(s) used to solve structure: *SHELXS97* (Sheldrick, 2008 ▶); program(s) used to refine structure: *SHELXL97* (Sheldrick, 2008 ▶); molecular graphics: *SHELXTL* (Sheldrick, 2008 ▶); software used to prepare material for publication: *SHELXL97*.

## Supplementary Material

Crystal structure: contains datablocks I, global. DOI: 10.1107/S1600536809000270/at2697sup1.cif

Structure factors: contains datablocks I. DOI: 10.1107/S1600536809000270/at2697Isup2.hkl

Additional supplementary materials:  crystallographic information; 3D view; checkCIF report

## Figures and Tables

**Table 1:** Hydrogen-bond geometry (Å, °)

*D*—H⋯*A*	*D*—H	H⋯*A*	*D*⋯*A*	*D*—H⋯*A*
O1*W*—H1*WA*⋯O1^i^	0.842 (17)	2.05 (2)	2.835 (3)	154 (4)
O1*W*—H1*WB*⋯O2^ii^	0.852 (17)	1.91 (2)	2.731 (2)	161 (3)
O2*W*—H2*WA*⋯O1^iii^	0.871 (17)	2.001 (18)	2.870 (2)	175 (3)
O2*W*—H2*WB*⋯O1*W*^i^	0.795 (18)	2.21 (3)	2.920 (3)	148 (3)
O2*W*—H2*WB*⋯O4^i^	0.795 (18)	2.20 (3)	2.780 (3)	130 (3)

Symmetry codes: (i) 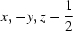; (ii) 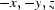; (iii) 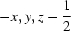.

**Fig. 1.**
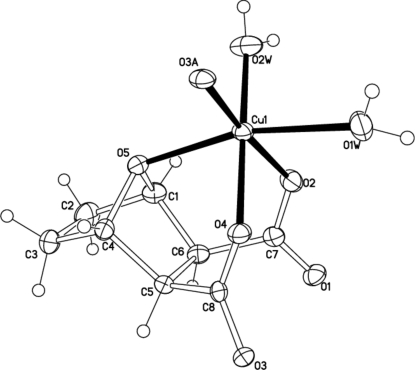
A view of the molecule of (I) showing the atom-labelling scheme with displacement ellipsoids drawn at the 30% probability.

## supplementary crystallographic information

### Comment

7-Oxabicyclo[2.2.1]heptane-2,3-dicarboxylic anhydride (norcantharidin), a traditional Chinese drug, has great anti-cancer activity. It has been widely used as an anticancer drug to treat hepatoma, lung cancer, esophagus cancer and gastric cancer for a long time. Copper is an essential microelement in human body and it exists in the form of copper proteins in animal bodies. Copper coordination compounds have strong bioactivity and various structures, therefore people pay more attention to them and have synthesized some complexes that have pronounced anticancer activity, bactericidal activity, anti-proliferative effect in recent years (Yin *et al.*, 2003). In order to prepare compounds with pronounced anti-cancer activity, we synthesized Cu^II^ complex of norcantharidin, whose anti-cancer activity test is being carried out.

In the title compound, each Cu^II^ ion is six-coordinated by two oxygen atoms from water, one bridge oxygen, two carboxylate oxygen atoms in two different carboxylate groups and one carboxylate oxygen atom in another asymmetric unit. O4, O5, O2W and O1W lie in the equatorial plane with the torsion angle -1.004 (62)°. Carboxylate oxygen atom O2 and O3 from another bridge ligand unit are in the axial positions. The bond angle of O2—Cu1—O3 is 171.256 (73)°, so it forms a distorted octahedral. Owing to the binding of the bridge oxygen atom with Cu, two six-membered rings (Cu1—O5—C4—C5—C8—O4 and Cu1—O2—C7—C6—C1—O5) are created. In addition, a seven-membered ring (Cu1—O4—C8—C5—C6—C7—O2) is formed because of the coordination of carboxylate oxygen atoms O2 and O4. What's more, intermolecular hydrogen bonds of the complex make the compound more stable.

### Experimental

A mixture of norcantharidin and CuCl_2._2H_2_O was dissolved in 20 mL absolute ethyl alcohol and stirred for 4 h at room temperature and then refluxed for 2 h at 333 K. The blue solution was filtered and after 2 weeks block green single crystals were obtained.

### Refinement

The H atoms bonded to C atoms were positioned geometrically and refined using a riding model [aliphatic C—H = 0.97 (2) Å, *U*_iso_(H) = 1.2*U*_eq_(C)]. The H atoms bonded to O atoms were located in a difference Fourier maps and refined with O—H distance restraints of 0.85 (2) and *U*_iso_(H) = 1.5*U*_eq_(O).

### Crystal data

**Table:**

[Cu(C_8_H_8_O_5_)(H_2_O)_2_]	*F*(000) = 1160
*M**_r_* = 283.72	*D*_x_ = 1.896 Mg m^−^^3^
Orthorhombic, *I**b**a*2	Mo *K*α radiation, λ = 0.71073 Å
Hall symbol: I 2 -2c	Cell parameters from 4186 reflections
*a* = 10.5512 (4) Å	θ = 2.1–27.5°
*b* = 19.3389 (9) Å	µ = 2.22 mm^−^^1^
*c* = 9.7435 (4) Å	*T* = 296 K
*V* = 1988.15 (14) Å^3^	Block, green
*Z* = 8	0.29 × 0.20 × 0.12 mm

### Data collection

**Table:**

Bruker APEXII area-detector diffractometer	2078 independent reflections
Radiation source: fine-focus sealed tube	1897 reflections with *I* > 2σ(*I*)
graphite	*R*_int_ = 0.021
ω scans	θ_max_ = 27.5°, θ_min_ = 2.1°
Absorption correction: multi-scan (*SADABS*; Sheldrick, 1996)	*h* = −13→13
*T*_min_ = 0.60, *T*_max_ = 0.78	*k* = −21→25
7372 measured reflections	*l* = −10→12

### Refinement

**Table:**

Refinement on *F*^2^	Secondary atom site location: difference Fourier map
Least-squares matrix: full	Hydrogen site location: inferred from neighbouring sites
*R*[*F*^2^ > 2σ(*F*^2^)] = 0.022	H atoms treated by a mixture of independent and constrained refinement
*wR*(*F*^2^) = 0.059	*w* = 1/[σ^2^(*F*_o_^2^) + (0.0368*P*)^2^] where *P* = (*F*_o_^2^ + 2*F*_c_^2^)/3
*S* = 1.02	(Δ/σ)_max_ < 0.001
2078 reflections	Δρ_max_ = 0.32 e Å^−^^3^
157 parameters	Δρ_min_ = −0.38 e Å^−^^3^
9 restraints	Absolute structure: Flack (1983), 857 Freidel pairs
Primary atom site location: structure-invariant direct methods	Flack parameter: 0.001 (16)

### Special details

**Table:**

Geometry. All e.s.d.'s (except the e.s.d. in the dihedral angle between two l.s. planes) are estimated using the full covariance matrix. The cell e.s.d.'s are taken into account individually in the estimation of e.s.d.'s in distances, angles and torsion angles; correlations between e.s.d.'s in cell parameters are only used when they are defined by crystal symmetry. An approximate (isotropic) treatment of cell e.s.d.'s is used for estimating e.s.d.'s involving l.s. planes.
Refinement. Refinement of *F*^2^ against ALL reflections. The weighted *R*-factor *wR* and goodness of fit *S* are based on *F*^2^, conventional *R*-factors *R* are based on *F*, with *F* set to zero for negative *F*^2^. The threshold expression of *F*^2^ > σ(*F*^2^) is used only for calculating *R*-factors(gt) *etc*. and is not relevant to the choice of reflections for refinement. *R*-factors based on *F*^2^ are statistically about twice as large as those based on *F*, and *R*- factors based on ALL data will be even larger.

### Fractional atomic coordinates and isotropic or equivalent isotropic displacement parameters (Å^2^)

**Table:**

	*x*	*y*	*z*	*U*_iso_*/*U*_eq_	
Cu1	0.243032 (19)	0.040771 (14)	0.45597 (9)	0.02570 (9)	
O1	0.08219 (15)	0.14342 (9)	0.76589 (19)	0.0351 (4)	
O1W	0.11498 (18)	−0.05770 (11)	0.4992 (2)	0.0476 (6)	
H1WA	0.117 (3)	−0.0923 (14)	0.447 (3)	0.071*	
H1WB	0.044 (2)	−0.0648 (17)	0.538 (3)	0.071*	
O2	0.12635 (15)	0.09609 (10)	0.5661 (2)	0.0367 (5)	
O2W	0.16510 (19)	0.08276 (13)	0.2897 (2)	0.0471 (6)	
H2WA	0.092 (2)	0.1034 (16)	0.283 (4)	0.071*	
H2WB	0.170 (3)	0.0631 (18)	0.218 (3)	0.071*	
O3	0.34512 (15)	0.02601 (8)	0.85851 (18)	0.0285 (4)	
O4	0.31828 (14)	0.00547 (9)	0.63735 (17)	0.0287 (4)	
O5	0.38661 (12)	0.13101 (8)	0.4694 (2)	0.0278 (3)	
C1	0.3288 (2)	0.19244 (12)	0.5275 (3)	0.0324 (6)	
H1A	0.2597	0.2112	0.4717	0.039*	
C2	0.4418 (3)	0.24140 (14)	0.5415 (3)	0.0458 (7)	
H2A	0.4284	0.2749	0.6142	0.055*	
H2B	0.4588	0.2655	0.4562	0.055*	
C3	0.5491 (2)	0.19062 (15)	0.5777 (3)	0.0421 (7)	
H3A	0.6159	0.1915	0.5092	0.051*	
H3B	0.5851	0.2007	0.6671	0.051*	
C4	0.4799 (2)	0.12166 (13)	0.5777 (3)	0.0284 (5)	
H4A	0.5357	0.0818	0.5635	0.034*	
C5	0.39748 (19)	0.11620 (12)	0.7068 (2)	0.0242 (5)	
H5A	0.4444	0.1336	0.7865	0.029*	
C6	0.2864 (2)	0.16753 (12)	0.6698 (3)	0.0269 (5)	
H6A	0.2863	0.2065	0.7343	0.032*	
C7	0.1552 (2)	0.13317 (12)	0.6683 (3)	0.0268 (5)	
C8	0.35037 (18)	0.04365 (11)	0.7353 (3)	0.0221 (5)	

### Atomic displacement parameters (Å^2^)

**Table:**

	*U*^11^	*U*^22^	*U*^33^	*U*^12^	*U*^13^	*U*^23^
Cu1	0.02380 (12)	0.03211 (16)	0.02120 (16)	0.00285 (9)	−0.00032 (15)	−0.00532 (16)
O1	0.0333 (8)	0.0431 (10)	0.0290 (10)	0.0053 (8)	0.0067 (8)	−0.0028 (8)
O1W	0.0330 (9)	0.0613 (12)	0.0486 (16)	−0.0163 (8)	0.0057 (8)	−0.0157 (10)
O2	0.0241 (8)	0.0512 (11)	0.0347 (13)	0.0028 (8)	0.0018 (7)	−0.0157 (10)
O2W	0.0439 (10)	0.0700 (16)	0.0276 (12)	0.0251 (10)	−0.0055 (9)	−0.0087 (10)
O3	0.0294 (8)	0.0350 (9)	0.0210 (10)	−0.0072 (7)	−0.0016 (7)	0.0082 (8)
O4	0.0346 (8)	0.0295 (9)	0.0222 (9)	−0.0048 (7)	0.0009 (6)	−0.0027 (7)
O5	0.0289 (6)	0.0320 (8)	0.0226 (9)	−0.0002 (6)	0.0016 (7)	0.0006 (8)
C1	0.0382 (13)	0.0283 (12)	0.0307 (16)	0.0052 (10)	−0.0037 (11)	0.0056 (12)
C2	0.0594 (19)	0.0329 (15)	0.0451 (19)	−0.0131 (12)	0.0053 (14)	0.0066 (14)
C3	0.0381 (14)	0.0525 (17)	0.0358 (18)	−0.0196 (12)	0.0025 (12)	0.0060 (13)
C4	0.0240 (10)	0.0346 (13)	0.0267 (14)	−0.0013 (9)	0.0013 (9)	0.0051 (11)
C5	0.0227 (9)	0.0287 (13)	0.0211 (13)	−0.0014 (9)	−0.0027 (8)	0.0002 (10)
C6	0.0322 (10)	0.0243 (12)	0.0242 (13)	0.0040 (9)	0.0004 (10)	−0.0038 (11)
C7	0.0254 (10)	0.0273 (12)	0.0277 (14)	0.0076 (9)	−0.0022 (10)	0.0022 (11)
C8	0.0134 (8)	0.0285 (12)	0.0245 (14)	0.0024 (8)	0.0014 (8)	0.0003 (10)

### Geometric parameters (Å, °)

**Table:**

Cu1—O3^i^	1.9313 (16)	C1—C2	1.528 (4)
Cu1—O2	1.9524 (17)	C1—C6	1.535 (4)
Cu1—O2W	1.990 (2)	C1—H1A	0.9800
Cu1—O4	2.0542 (19)	C2—C3	1.540 (4)
Cu1—O5	2.3147 (14)	C2—H2A	0.9700
Cu1—O1W	2.3726 (19)	C2—H2B	0.9700
O1—C7	1.240 (3)	C3—C4	1.521 (3)
O1W—H1WA	0.842 (17)	C3—H3A	0.9700
O1W—H1WB	0.852 (17)	C3—H3B	0.9700
O2—C7	1.264 (3)	C4—C5	1.533 (3)
O2W—H2WA	0.871 (17)	C4—H4A	0.9800
O2W—H2WB	0.795 (18)	C5—C8	1.514 (3)
O3—C8	1.249 (3)	C5—C6	1.578 (3)
O3—Cu1^ii^	1.9313 (16)	C5—H5A	0.9800
O4—C8	1.253 (3)	C6—C7	1.536 (3)
O5—C1	1.450 (3)	C6—H6A	0.9800
O5—C4	1.454 (3)		
			
O3^i^—Cu1—O2	171.25 (8)	C3—C2—H2A	111.5
O3^i^—Cu1—O2W	95.92 (9)	C1—C2—H2B	111.5
O2—Cu1—O2W	87.90 (8)	C3—C2—H2B	111.5
O3^i^—Cu1—O4	89.15 (7)	H2A—C2—H2B	109.3
O2—Cu1—O4	87.30 (8)	C4—C3—C2	101.87 (19)
O2W—Cu1—O4	174.69 (8)	C4—C3—H3A	111.4
O3^i^—Cu1—O5	99.62 (6)	C2—C3—H3A	111.4
O2—Cu1—O5	88.19 (7)	C4—C3—H3B	111.4
O2W—Cu1—O5	90.51 (9)	C2—C3—H3B	111.4
O4—Cu1—O5	87.07 (6)	H3A—C3—H3B	109.3
O3^i^—Cu1—O1W	82.43 (8)	O5—C4—C3	102.49 (19)
O2—Cu1—O1W	89.03 (8)	O5—C4—C5	102.70 (16)
O2W—Cu1—O1W	103.70 (9)	C3—C4—C5	109.4 (2)
O4—Cu1—O1W	78.49 (7)	O5—C4—H4A	113.7
O5—Cu1—O1W	165.41 (8)	C3—C4—H4A	113.7
Cu1—O1W—H1WA	121 (2)	C5—C4—H4A	113.7
Cu1—O1W—H1WB	135 (2)	C8—C5—C4	113.6 (2)
H1WA—O1W—H1WB	99 (2)	C8—C5—C6	112.40 (17)
C7—O2—Cu1	126.29 (15)	C4—C5—C6	100.98 (19)
Cu1—O2W—H2WA	128 (3)	C8—C5—H5A	109.9
Cu1—O2W—H2WB	120 (3)	C4—C5—H5A	109.9
H2WA—O2W—H2WB	102 (2)	C6—C5—H5A	109.9
C8—O3—Cu1^ii^	132.82 (15)	C1—C6—C7	112.9 (2)
C8—O4—Cu1	124.31 (15)	C1—C6—C5	100.79 (19)
C1—O5—C4	95.93 (17)	C7—C6—C5	113.56 (18)
C1—O5—Cu1	111.39 (12)	C1—C6—H6A	109.7
C4—O5—Cu1	112.98 (13)	C7—C6—H6A	109.7
O5—C1—C2	102.40 (19)	C5—C6—H6A	109.7
O5—C1—C6	102.60 (18)	O1—C7—O2	123.0 (2)
C2—C1—C6	110.0 (2)	O1—C7—C6	118.9 (2)
O5—C1—H1A	113.6	O2—C7—C6	118.1 (2)
C2—C1—H1A	113.6	O3—C8—O4	124.0 (2)
C6—C1—H1A	113.6	O3—C8—C5	116.3 (2)
C1—C2—C3	101.5 (2)	O4—C8—C5	119.7 (2)
C1—C2—H2A	111.5		
			
O2W—Cu1—O2—C7	−131.2 (2)	C2—C3—C4—O5	34.5 (2)
O4—Cu1—O2—C7	46.5 (2)	C2—C3—C4—C5	−74.0 (3)
O5—Cu1—O2—C7	−40.6 (2)	O5—C4—C5—C8	86.0 (2)
O1W—Cu1—O2—C7	125.0 (2)	C3—C4—C5—C8	−165.65 (19)
O3^i^—Cu1—O4—C8	139.29 (16)	O5—C4—C5—C6	−34.5 (2)
O2—Cu1—O4—C8	−48.70 (17)	C3—C4—C5—C6	73.8 (2)
O5—Cu1—O4—C8	39.62 (16)	O5—C1—C6—C7	−85.9 (2)
O1W—Cu1—O4—C8	−138.26 (17)	C2—C1—C6—C7	165.7 (2)
O3^i^—Cu1—O5—C1	174.10 (15)	O5—C1—C6—C5	35.6 (2)
O2—Cu1—O5—C1	−9.87 (16)	C2—C1—C6—C5	−72.8 (2)
O2W—Cu1—O5—C1	78.01 (16)	C8—C5—C6—C1	−122.0 (2)
O4—Cu1—O5—C1	−97.26 (16)	C4—C5—C6—C1	−0.6 (2)
O1W—Cu1—O5—C1	−89.0 (3)	C8—C5—C6—C7	−0.9 (3)
O3^i^—Cu1—O5—C4	−79.25 (15)	C4—C5—C6—C7	120.5 (2)
O2—Cu1—O5—C4	96.78 (15)	Cu1—O2—C7—O1	−148.85 (19)
O2W—Cu1—O5—C4	−175.34 (15)	Cu1—O2—C7—C6	31.8 (3)
O4—Cu1—O5—C4	9.39 (14)	C1—C6—C7—O1	−142.1 (2)
O1W—Cu1—O5—C4	17.7 (3)	C5—C6—C7—O1	104.0 (3)
C4—O5—C1—C2	56.4 (2)	C1—C6—C7—O2	37.3 (3)
Cu1—O5—C1—C2	173.91 (15)	C5—C6—C7—O2	−76.7 (3)
C4—O5—C1—C6	−57.6 (2)	Cu1^ii^—O3—C8—O4	−25.2 (3)
Cu1—O5—C1—C6	59.9 (2)	Cu1^ii^—O3—C8—C5	154.10 (15)
O5—C1—C2—C3	−34.9 (3)	Cu1—O4—C8—O3	148.71 (18)
C6—C1—C2—C3	73.6 (3)	Cu1—O4—C8—C5	−30.6 (2)
C1—C2—C3—C4	0.2 (3)	C4—C5—C8—O3	142.9 (2)
C1—O5—C4—C3	−56.3 (2)	C6—C5—C8—O3	−103.2 (2)
Cu1—O5—C4—C3	−172.55 (15)	C4—C5—C8—O4	−37.8 (3)
C1—O5—C4—C5	57.25 (19)	C6—C5—C8—O4	76.1 (3)
Cu1—O5—C4—C5	−59.00 (19)		

Symmetry codes: (i) *x*, −*y*, *z*−1/2; (ii) *x*, −*y*, *z*+1/2.

### Hydrogen-bond geometry (Å, °)

**Table:**

*D*—H···*A*	*D*—H	H···*A*	*D*···*A*	*D*—H···*A*
O1W—H1WA···O1^i^	0.84 (2)	2.05 (2)	2.835 (3)	154 (4)
O1W—H1WB···O2^iii^	0.85 (2)	1.91 (2)	2.731 (2)	161 (3)
O2W—H2WA···O1^iv^	0.87 (2)	2.00 (2)	2.870 (2)	175 (3)
O2W—H2WB···O1W^i^	0.80 (2)	2.21 (3)	2.920 (3)	148 (3)
O2W—H2WB···O4^i^	0.80 (2)	2.20 (3)	2.780 (3)	130 (3)

Symmetry codes: (i) *x*, −*y*, *z*−1/2; (iii) −*x*, −*y*, *z*; (iv) −*x*, *y*, *z*−1/2.
